# Electronic States of Tris(bipyridine) Ruthenium(II) Complexes in Neat Solid Films Investigated by Electroabsorption Spectroscopy

**DOI:** 10.3390/ma15062278

**Published:** 2022-03-19

**Authors:** Daniel Pelczarski, Oleksandr Korolevych, Błażej Gierczyk, Maciej Zalas, Małgorzata Makowska-Janusik, Waldemar Stampor

**Affiliations:** 1 Department of Molecular Photophysics, Institute of Applied Physics and Mathematics, Gdańsk University of Technology, Narutowicza 11/12, 80-233 Gdańsk, Poland; 2 Faculty of Science and Technology, Jan Długosz University, Armii Krajowej 13/15, 42-200 Częstochowa, Poland; oleksandr.korolevych@doktorant.ujd.edu.pl (O.K.); m.makowska@ujd.edu.pl (M.M.-J.); 3 Faculty of Chemistry, Adam Mickiewicz University, Uniwersytetu Poznańskiego 8, 61-614 Poznań, Poland; hanuman@amu.edu.pl (B.G.); maciej.zalas@amu.edu.pl (M.Z.)

**Keywords:** electroabsorption spectroscopy, ruthenium dyes, polypyridine complexes, metal-to-ligand charge transfer, Stark effect, quantum chemical simulations

## Abstract

We present the electric field-induced absorption (electroabsorption, EA) spectra of the solid neat films of tris(bipyridine) Ru(II) complexes, which were recently functionalized in our group as photosensitizers in dye-sensitized solar cells, and we compare them with the results obtained for an archetypal [Ru(bpy)_3_]^2+^ ion (RBY). We argue that it is difficult to establish a unique set of molecular parameter values by discrete parametrization of the EA spectra under the Liptay formalism for non-degenerate excited states. Therefore, the experimental EA spectra are compared with the spectra computed by the TDDFT (time-dependent density-functional theory) method, which for the first time explains the mechanism of electroabsorption in tris(bipyridine) Ru complexes without any additional assumptions about the spectral lineshape of the EA signal. We have shown that the main EA feature, in a form close to the absorption second derivative observed in the spectral range of the first MLCT (metal-to-ligand charge transfer) absorption band in Ru(bpy)_3_(PF_6_)_2_, can be attributed to a delocalized and orbitally degenerate excited state. This result may have key implications for the EA mechanism in RBY-based systems that exhibit similar EA spectra due to the robust nature of MLCT electronic states in such systems.

## 1. Introduction

Organometallic ruthenium(II) complexes in general and polypyridine Ru(II) complexes in particular constitute an important group of materials with many applications as solar energy sensitizers and photoredox agents, in dye-sensitized solar cells (DSSCs) that efficiently convert sunlight to electricity, and in photoelectrochemical water-splitting devices that produce environmentally friendly hydrogen fuel. The versatile utility of Ru(II) complexes results mainly from their robust MLCT excited states involved in optical absorption and emission processes. The MLCT states of Ru(II) complexes, in which a Ru 4d-electron is transferred to π* orbitals of ligands, have been the subject of numerous experimental and theoretical investigations during the last five decades (for a literature survey on this subject, see [1,2,3,4,5,6,7]). One of the basic issues in understanding the electronic properties of this type of complex is the degree of spatial charge delocalization in the excited states resulting from the metal-to-ligand charge transfer.

A prototypical complex in this group is the trischelate [Ru(II)(bpy)_3_]^2+^ ion (hereinafter referred to as RBY) with 2,2′-bipyridine (bpy) ligands. The ground state of this ion is a singlet state with the D_3_ symmetry of a three-blade propeller. The electronic transitions to the lowest energy singlet MLCT states of this complex are ascribed to the main absorption band in the visible spectral range. In room temperature liquid and solid solutions, this absorption band is rather broad, with some only weakly resolved spectral features derived from multiple electronic transitions and possible contributions of various vibrational modes of the molecule. The question has long been raised as to whether the photo-excited state detected in absorption is localized only on one of the bpy ligands, [Ru(III)(bpy^−^)(bpy)_2_]^2+^, breaking symmetry of the ground state, or it is spread over three equivalent ligands, [Ru(III)(bpy^1/3−^)_3_]^2+^, preserving the D_3_ ground state symmetry. After being initially prepared on an electronic timescale of 10^−15^ s, the singlet excited state, ^1^MLCT, due to strong spin-orbit coupling (SOC) of Ru core electrons, rapidly (≅30 fs [8]) and with a quantum yield near unity undergoes intersystem crossing to the triplet manifold, ^3^MLCT, which is involved in emission processes. Many sophisticated time-resolved (on femtosecond to nanosecond time scales) spectroscopy experiments have been carried out to unravel the complex relaxation phenomena of electronic and vibrational structures during the formation of emitting long-lived ^3^MLCT states. Therefore, it is well established and confirmed by quantum chemical TDDFT calculations that the relaxed emissive ^3^MLCT state in a liquid solution, glassy, or nonsymmetrical crystal matrix is localized on one Ru-bpy segment of a C_2_ geometry molecule only slightly changed from the ground-state geometry D_3_ [9].

The situation is not so clear-cut if we consider absorption processes and the properties of the initially excited (Franck–Condon) states of a RBY complex. Now, it is necessary to take into account the interaction of radiation field with the symmetrical (D_3_) ground state of the complex composed of three structurally equivalent ligands. If radiation interacts equivalently with all three ligands, assuming the validity of the adiabatic approximation, the vertically excited state of the free ion is spread symmetrically over the whole complex. If only electronic interactions are considered, both the ground and MLCT excited singlet states are certainly systems with symmetrically delocalized electron distributions. Since in a D_3_ point group, the totally symmetric irreducible representation (A_1_) does not transform as x, y, or z coordinates, this inevitably means there is a lack of permanent electric dipole moments of the D_3_ molecular entity for all its energy levels including, beyond A_1_, the other two representations, A_2_ and E, in a D_3_ point group: [10]. The latter fact was confirmed by spectroscopic studies of the complex in the gas-phase [11]. If the RBY ion is embedded in a symmetrical crystal matrix, the main absorption band of singlet MLCT parentage is dominated by orbitally doubly-degenerate excited states of E symmetry with two sub-components (x, y)-polarized in the plane perpendicular to the z-direction of the C_3_ axis [12].

Experiments on the photodissociation spectroscopy of mass selected solvent adducts of RBY revealed that attaching a single polar acetonitrile (ACN) molecule to the Ru complex did not significantly shift the MLCT band of the photodissociation action spectrum in the visible spectral range, suggesting that the relevant MLCT excited states are initially delocalized with no permanent electric dipole moment [13]. This interpretation is consistent with earlier femtosecond absorption anisotropy measurements in nitrile solutions [14], where it was found that the initial delocalization of the excited state over all three ligands in a coupled RBY-solvent system is followed by charge localization onto a single ligand due to solvent reorganization. In principle, changes in the permanent dipole moment upon electron excitation can be induced by electronic interaction with a nonsymmetric environment. However, due to structural rigidity of the complex and robust character of degenerate E states, the relevant changes are difficult to observe [15]. 

The solvatochromism of a RBY complex in liquid solvents is rather weak, and the solvent shifts of the MLCT absorption band [16] are similar in size to the higher-energy (^1^B_2u_) singlet state for the nonpolar anthracene molecule [17] but rather quite small when compared to typical molecular charge-transfer bands [2]. Although the observed solvent dependence of the MLCT absorption band by Kober et al. [16] was ascribed to the polar excited state with an electron localized on a single ligand and a permanent dipole moment of 14 D, this interpretation was later criticized for several reasons [2,15,18]. The absorption spectra of a related symmetric complex, [Ru(dcebpy)_3_]^2+^ (where dcebpy = 4,4′-dicarboethoxy-2,2′-bipyridine) in solvents, covering a range of dielectric constants, displayed as opposed to ref. [16] nonsystematic shifts, which were tentatively attributed to specific solvent effects on a delocalized unpolar MLCT state [19]. The simplified formula for the solvent shift used in ref. [16] was based on dipole approximation in a continuum polarization medium and neglected the orbital degeneracy of E-type states and all terms responsible for the dispersion and higher multipole interactions for nonpolar solutes. The solvatochromism in the absorption and emission spectra of highly symmetric multipolar and dendrimer systems was analyzed in the context of the charge instability of primary nonpolar excited states and breaking their symmetry during the relaxation process, leading to polar emissive states (see e.g., [20]). Recently, the authors of ref. [15] repeated the original experiments for an RBY complex [16] and in terms of a more comprehensive Liptay’s description of solvatochromism in a polarizable continuous medium [21] (but neglecting dispersion and higher multipole terms) received a permanent dipole moment for an MLCT state a factor of 2 lower than Kober et al. [16]. More importantly, TDDFT quantum–chemical calculations performed for solute–solvent clusters containing up to 18 explicit solvent molecules placed in a solvent continuum indicate at the non-vanishing permanent dipole moments of both the ground and excited MLCT state. The dipole moments are hypothetically induced by the short-range unsymmetrical interactions of the solute, in which the closest solvent molecules penetrate the solute structure provided the electron polarizability of the chromophore is different in the ground and excited state. However, the D_3_ symmetry is only slightly distorted in the solvent environment, but the effect is, according to the TDDFT calculations performed, sufficient to split the energy levels and to twist the dipoles of the two components from their original opposite orientation in the doubly degenerate MLCT state of the complex in the gas phase [15].

The most suitable method, specifically dedicated to tracking charge redistribution immediately after photoexcitation, is electroabsorption (EA) spectroscopy, which is also known as Stark effect spectroscopy [22,23]. When an external electric field is applied to the molecular system, the absorption bands in general broaden, shift, and change in intensity. Using the standard EA spectral analysis, based on Liptay theory [24] for well-separated and non-degenerate electronic states, the total electro-optic effect can be quantified in terms of molecular parameters. Thus, changes in the permanent dipole moment (Δμ), electronic polarizability (Δ*p*), and the oscillator strength can be deduced by comparing the observed EA spectra with the derivatives (with respect to the photon energy) of the second, first, and zeroth order of the ordinary absorption spectrum of the molecular species under study, respectively. Indeed, this method was applied to many organometallic complexes embedded in polymer or glassy matrix (e.g., [25,26]). In our group, we investigated the solid neat films of the archetypal complexes in organic electronics such as 8-hydroxyquinoline aluminum(III) complex (Alq_3_) [27], 2-phenylpyridine iridium(III) complex (Ir(ppy)_3_) [28], and Pt(II) octaethylporphyrin (PtOEP) [29], to mention a few examples. The results for Alq_3_ indicate the lowest excited singlet states of the complex with relatively small permanent dipole moments, which are well localized on individual quinolate ligands, and those with higher energy and large dipole moments show delocalization by charge transfer to the nearest ligands of neighboring molecules. However, the latter effect is insignificant in the case of Ir(ppy)_3_ solid films where the lowest excited states of MLCT character are delocalized on the three ppy ligands and are endowed with a permanent dipole moment directed along the C_3_ symmetry axis of the molecule. An interesting case is the electronic states delocalized within a porphyrin ring in the PtOEP complex of four-fold symmetry, which are orbitally degenerate (of E_u_ symmetry in the D_4h_ point group) and behave in the electric field as Frenkel-type neutral excitons, preserving a symmetrical charge distribution.

According to standard (Liptay) description [24], two limiting cases can be distinguished. In more electronically delocalized molecular systems, the electron polarizability term usually dominates, and the EA spectrum should reproduce the first derivative of the absorption spectrum, while in more localized systems with an asymmetric charge distribution and a non-zero permanent dipole moment, the EA signal should mimic the second derivative of the absorption spectrum (confer Formula (4)). The latter observation is often taken as evidence for excited-state polarity, and therefore, EA spectroscopy is considered as a “direct” method to measure the permanent dipole change after photoexcitation. Of course, this is only true within the constraints of Liptay theory. 

Two sets of EA data for the RBY complex are available in the literature [30,31]; both were interpreted using Liptay formalism in terms of second derivative(s) of the absorption spectrum. In the pioneering work by Oh and Boxer [30] for a RBY(PF_6_)_2_ dissolved in polyvinyl alcohol (PVA) and measured at 77 K, two polar electronic states with permanent dipole moment changes of about 9 D/*f* and 5 D/*f* were recognized in the first MLCT manifold absorption band in order of increasing photon energy (*f* is a local electric field correction factor). In ref. [31], for a RBY(Cl)_2_⋅6H_2_O adduct in a PVA matrix at room temperature, in addition to the two above-mentioned states, the third electronic state in the lower energy spectral range with a circa two to three times smaller permanent dipole was also postulated. Although the authors of both articles assigned the electronic states active in EA as singlet orbitally degenerate E states, they inconsistently interpret the received dipole moments in terms of electron transfer from the Ru center to a single ligand, thus assuming a localized model of MLCT states. 

The EA spectroscopy is a useful method sensitive to the presence of the molecular excitations that are difficult to observe in other spectroscopies. However, interpretation of the EA spectra is a delicate matter. Comparing the EA spectrum with derivatives of the absorption spectrum is not always adequate to reliably describe the electronic transitions studied. A very characteristic and instructive example is presented in ref. [32], where two quasi-degenerate (accidentally) excited states, individually giving the lineshape of the first derivative of the absorption spectrum, produce in total a second-derivative EA signal, although neither of the states is endowed with a permanent dipole moment. The next important example is an orbitally degenerate (by symmetry) electronic state as in s-triazine where the first-order (linear) Stark effect should be considered [33]. The electroabsorption behavior of orbitally degenerate E states of molecular systems of C_3_ symmetry was thoroughly analyzed in the literature [34,35,36]. In short, when an excited state is degenerate, the energy level of this eigenstate is split in the electric field into two sublevels and, consequently, the absorption spectrum is also split into two sub-bands. The magnitude of the splitting, $2{\vec{\mu }}_{12}\cdot \vec{F}$, is determined by the dipole moment matrix element ${\vec{\mu }}_{12}$ (in symmetry adapted basis) expressing coupling between sublevels 1 and 2. When the bandwidth exceeds the splitting, instead of splitting, we usually observe an electric field-induced broadening of the absorption spectrum with the EA signal exhibiting the second-derivative lineshape. It would be practically impossible to discern experimentally between this mechanism and that involving a permanent dipole moment, which was developed for non-degenerate systems. An important message is that an excited degenerate state without a permanent dipole moment can give rise to the EA signal of the second-derivative lineshape with a scaling factor dependent on the dipole coupling between the respective components of the degenerate state (μ_12_). The detailed quantitative analysis requires taking into account the degree of electronic delocalization of the trimer molecular systems considered [34,35]. 

The standard Liptay formalism [24] can also be difficult to apply when the absorption spectra are highly congested by electronic/vibronic states, which is also the case with the RBY complex. In the spectral range of 6000 cm^−1^ of the first MLCT absorption band of the complex, at least nine singlet and 27 triplet states are involved [2,4], not counting their various vibrational modes with different vibronic coupling mechanisms. The coexistence of multiple excited states with possibly different properties implies that the overall EA response of the molecular system is a function of too many parameters to be unambiguously estimated, as already noted by Liptay himself [37]. To solve the problem, the EA spectra should be rather determined directly by definition as the difference between the absorption spectra computed in the nonzero and in the zero field without arbitrary assumptions as to the shape of the individual EA features (of first or second derivative). The Petelenz group [38] applied this approach very successfully in reproducing the experimental EA spectra of polyacene solid films. In their microscopic model of electroabsorption, the interaction with the modulating electric field is explicitly included in the Hamiltonian, and only the input parameters are defined and estimated in terms of molecular wave functions. Some progress has been as well achieved in taking into account the vibronic coupling mechanism in EA behavior of poly-nuclear Ru complexes of mixed valence properties, although the more tractable description is at best restricted here to one electronic transition coupled to one or two vibrational modes [39,40].

**Figure 1:**
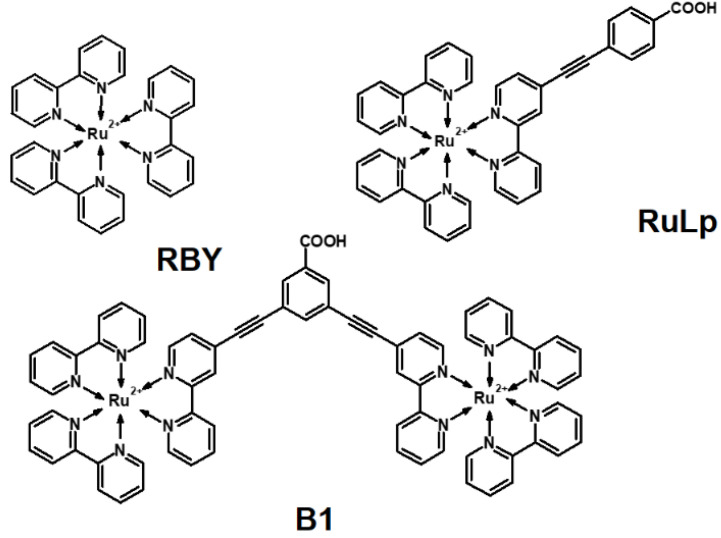
The molecular structures of Ru(II) complexes.

In this article, we present the EA spectra of solid neat films for RBY(PF_6_)_2_ and two other Ru(II) complexes: RuLp and B1 (for molecular structures see Figure 1), which were recently functionalized in our group [41,42] as photosensitizers in DSSCs. Our EA results for neat solid films are compared with the literature data for RBY(PF_6_)_2_ and RBY(Cl)_2_⋅6H_2_O species dissolved in glassy or polymer, which are naturally more disordered environments to capture the possible factors responsible for the EA mechanism. Particular attention was paid for the degeneracy or quasi-degeneracy of the respective excited states and its influence on the spectral shape of the EA signal and the polarity of the excited states. We argue that it is difficult to establish a unique set of molecular parameter values by the discrete parametrization of EA spectra under the Liptay formalism for non-degenerate excited states. Therefore, the experimental EA spectra have been compared with the spectra computed by the TDDFT method, which for the first time explains the mechanism of electroabsorption in tris(bipyridine) Ru complexes without additional assumptions about the molecular parameters of the excited states. The main feature of the EA signal observed in the spectral range of the first MLCT band in RBY(PF_6_)_2_, clearly displaying the shape of the second derivative of the absorption spectrum, can be reasonably rationalized as a fingerprint of a delocalized and orbitally degenerate excited state.

## 2. Electroabsorption

An electric field ($\vec{F}$) changes the absorption spectrum of a molecule or set of molecules with a fixed (and independent of electric field) orientation by the shift, Δ*E*($\vec{F}$), of the energy (*E*), of a single electronic transition, due to the Stark effect,

(1) $$\Delta E\left(\vec{F}\right)=-\vec{\Delta \mu }\cdot \vec{F}-\frac{1}{2}\vec{F}\circ \Delta p\circ \vec{F}\;,$$

where $\vec{\Delta \mu }$ is the change in permanent dipole moment and Δ**p** is the change in electronic polarizability tensor after transition from the ground state to the excited non-degenerate state. If the field-induced change (Δ*D*) of the absorbance (optical density *D*) is sufficiently small, it can be expanded in a Maclaurin series with respect to energy and truncated beyond the quadratic term. Assuming that the oscillator strength of the electronic transition is independent of the electric field, the measured values of Δ*D* (*E*, *F*) can be related with the averaged shift <Δ*E*> and the averaged broadening <(Δ*E*)^2^> of the absorption band in the isotropic ensemble of molecules [43]:

(2) $$<\Delta E>=-\frac{1}{2}\bar{\Delta p}\;F^{2}\;,$$

(3) $$<{\left(\Delta E\right)}^{2}>=\kappa {\left(\Delta \mu \right)}^{2}\;F^{2}\;,$$

where the value of the κ coefficient depends on the exact form of the averaging procedure (see below). Thus, the average of Δ*D* is given by

(4) $$\Delta D=\left(\;\frac{1}{2}\;\bar{\Delta p}\;\frac{dD}{dE}+\kappa \;{\left(\Delta \mu \right)}^{2}\frac{d^{2}D}{dE^{2}}\right)\cdot F^{2}=B\left(E\right)\cdot F^{2}\;.$$

Here, *B* stands for the function expressing the spectral dependence of Δ*D*. Formula (4) was derived for a beam of non-polarized light incident perpendicularly on a molecular layer equipped with sandwich electrodes, which means the angle between the direction of the light beam (polarization) electric field and the external applied electric field χ = 90°. According to Liptay’s description [24], the averaging factor κ generally depends on the angle ξ between the transition dipole moment and the permanent dipole moment change (Δμ), κ = (2 − cos^2^ξ)/5 which for pure CT or MLCT states with the dominant so-called transfer term [44] is equal to 1/5 (ξ = 0). Although the description of the electronic degenerate state, based on the linear Stark effect, has a slightly different physical mechanism, the final Formula (4) for the electroabsorption signal Δ*D* is still valid, but in this case, it is κ = 3/10, and instead of Δμ, one should put the dipole moment matrix element (μ_12_) between two components that form the electronic state [34]. It is worth noting that the less stringent averaging method gives the same value of κ = 1/3 in both cases [36,43] and almost equal to κ = 3/10 for strictly degenerate electronic states (confer Table 1). In this article, we will take κ = 1/3 to evaluate and compare Δμ in all the cases considered. The underlying assumption that the oscillator strength is electric field independent is usually satisfied in molecular systems (for Ru complexes, see, e.g., [25,26]) as long as the electronic wave functions are well localized within the molecular dimension. 

Basically, a direct comparison of the EA spectrum with the absorption derivatives based on Formula (4) should allow us to extract information about dipole moment (Δμ) and polarizability $\bar{\Delta p}$ changes. However, as mentioned in the Introduction and discussed later, when multiple excited states in a narrow spectral range are involved, the EA spectrum requires careful and thorough analysis. 

## 3. Materials and Methods

### 3.1. Materials and Experimental Details

The Ru(II) complexes RBY, RuLp, and B1 (Figure 1) with hexafluorophosphate (${\mathrm{PF}}_{6}^{-}$) counterions were investigated. In RuLp, the *p*-carboxyphenyl group is attached via an ethynyl bridge to one of the bpy ligands of the RBY unit, and in the biruthenium B1 complex, two RBY units are linked by ethynyl bridges to an electronic carboxybenzene coupler. From the starting materials, RBY(PF_6_)_2_, RuLp(PF_6_)_2_, and B1(PF_6_)_4_, which were prepared and purified as described previously [41,42], the solid neat films by the spin-coating method were formed. Spinning a solution of a Ru material in dichloromethane with a speed of 1000 rpm allowed obtaining smooth films of 40–60 nm thickness. The thickness was measured with a Tencor Alpha Step 500 Profiler. 

SEM (scanning electron microscopy) images of the obtained solid films did not reveal crystallites with a scan resolution of several dozen nanometers; moreover, no long-range layering of molecules was found based on X ray scattering measurements for RBY(PF_6_)_2_ films [45]. Unlike solid Ir(ppy)_3_ films displaying crystalline microdomains [28] and similar to Alq_3_ films [27], produced by vacuum deposition, the spin-coated Ru complex solid films exhibit a quasi-amorphous structure. This is not unique but rather typical for organic films manufactured under non-thermodynamic conditions [46,47]. The term quasi-amorphous here means that the long-range order typical of single crystal is absent, but the molecular packing mimics the crystal state in the short range. Therefore, the degree of intermolecular ordering is strongly dependent on the deposition method, where the spin-casting deposition method leads to a lower order [48]. Accordingly, it can be assumed that the local D_3_ symmetry of an RBY ion in the crystal structure of RBY(PF_6_)_2_ [49] is also preserved in solid films. In addition, statistical fluctuations of molecular coordinates in solid films lead usually to the energy distribution of localized states, which can be recognized in the relevant broadening of the exciton bands observed in absorption spectra.

The ordinary absorption spectra (with the *D* absorbance ordinate) were recorded with a Perkin-Elmer Lambda 10 spectrophotometer. The electroabsorption was measured in a sandwich cell, quartz/Al/organic film/Al, with two vacuum-evaporated semi-transparent Al electrodes. A light beam from a halogen or xenon lamp, dispersed by a grating monochromator (Acton Research, model SP-2150), passed perpendicularly through the sample. As a detector, a Si photodiode (Hamamatsu S1337-66BR) or a low-gain photomultiplier (EMI 9781R) was used in the VIS and UV spectral ranges, respectively. The EA signals, which were recorded at the second (2ω) harmonic of frequency of the modulating sinusoidal electric field (ω) applied to the sample, are defined by

(5) $$\mathrm{EA}\;\equiv \;\frac{I_{2\omega }}{I_{0\omega }}\;,$$

where $I_{2\omega }$ stands for the rms value of the (2ω)-Fourier component, which was measured with a lock-in amplifier (Princeton Applied Research, model 5210), and $I_{0\omega }$ stands for the value of the (0ω)-Fourier component of transmitting light *I*, which was probed with an electrometer (Keithley, model 2000). All measurements were fully computer controlled, and EA signals as low as 10^−6^ could be measured. 

The directly measured EA signals, defined by Formula (5), can be related to the ΔD signals in Formula (4), which were preferred by other authors, by

(6) $$\mathrm{EA}=\frac{\ln\left(10\right)}{2\;\sqrt{2}}\;F_{e}{}^{2}\cdot B\left(E\right)=\;\frac{\ln\left(10\right)}{2\;\sqrt{2}}\;\Delta D.$$

In Formula (6), *F*_e_ = *f U*/*d*, where *f* is the local field correction factor and *U* is the external voltage amplitude applied to the sample of thickness *d*. No good method has been available to experimentally determine the local electric field, and the correction factor *f* can be approached in various ways. In the *Lorentz approximation*, often applied in organic molecular solids, the molecule is placed in a spherical vacuum cavity created in a dielectric continuous medium; then, *f* = (ε + 2)/3, and for a typical relative permittivity, ε = 3–4, we obtain the value *f* = 1.7–2.0. In turn, in the *applied field approximation*, *f* = 1 is usually assumed.

### 3.2. Numerical Calculations

The first D1 (d*D*/d*E*) and second D2 (d^2^*D*/d*E*^2^) derivatives of the measured absorption spectra were calculated using the Savitzky–Golay method (Origin 2020b program). The derivative spectra were smoothed by local polynomial (fifth-order) regression around each point using a Savitzky–Golay filter. The absorption spectra were decomposed ($D=\underset{n}{\sum }D_{n}$) into several Gaussian profiles,

(7) $$D_{n}=D_{0n}\;\exp\left[-4\;\ln2\;{\left(E-E_{n}\right)}^{2\;}/w_{n}{}^{2}\;\right]\;,$$

where *w_n_* = FWHM (full width at half maximum), *E_n_* is the center position, and *D*_0*n*_ is the maximal intensity (height) of the *n*th Gaussian band.

The measured EA spectra should be described by a sum of EA responses from all transitions occurring in a given spectral range. Based on the Liptay formalism (see Section 2), the EA spectrum can be decomposed into a linear combination of derivatives of Gaussian bands. Therefore, the EA experimental data were fit with theoretical curves calculated according to Equation (8) or Equation (9) for model 1:

(8) $$\mathrm{EA}=\underset{n}{\sum }b_{n}\;\frac{d^{2}D_{n}}{dE^{2}}\;,$$

for model 2:

(9) $$\mathrm{EA}=a\;\frac{dD}{dE}+\underset{n}{\sum }b_{n}\;\frac{d^{2}D_{n}}{dE^{2}}\;.$$

In the first approach (model 1), the dominant contribution of second-derivative components, associated with the permanent dipole moments (Δμ*_n_*), was assumed. In model 2, the first derivative (d*D*/d*E*) of the total (experimental) absorption spectrum (*D*) with a single scaling factor (*a*) was added, assuming the same electronic polarizability change (Δ*p*) for all transitions considered. The fitting procedure was performed using a non-linear least-squares method based on the Levenberg–Marquardt algorithm [50]. The algorithm determined the best-fit parameters through iterative minimization of a χ^2^ merit function using the gradient method. The fitting parameters were coefficients, *a* and *b_n_*, energy positions (*E_n_*), and bandwidths (*w_n_*) of Gaussian bands. From the best fit values of *a* and *b_n_*_,_ taking the intensities of individual Gaussians from the ordinary absorption spectrum, the polarizability (Δ*p*) and dipole moment changes (Δμ*_n_*) were evaluated. 

In the special case of a well-separated electronic transition, Equation (8) can be converted to a simple formula that allows evaluating the Δμ*_n_* (in debye unit, 1D = 3.34·10^−30^ C·m):

(10) $$\Delta \mu_{n}=2.8\cdot 10^{4}\;{\left(\frac{{\mathrm{EA}}_{\max}}{\kappa \;D_{0n}}\right)}^{\frac{1}{2}}\frac{w_{n}}{F_{\mathrm{rms}}}\;.$$

This formula is valid for a Gaussian band with amplitude *D*_0*n*_, width *w_n_* (in cm^−1^), and the maximum electroabsorption signal EA_max_ obtained in an electric field with the rms value *F*_rms_ (in V/cm).

For convenience, the abscissa of absorption and EA spectra is expressed as the wavenumber in units of kilokaysers (1 kK = 10^3^ cm^−1^).

### 3.3. Quantum Chemical Calculations

When starting the analysis of the electronic properties of the RBY(PF_6_)_2_ molecule, its singlet ground state geometry in a free space was first optimized by the DFT method using B3LYP exchange-correlation potentials in generalized gradient approximation (GGA) with the hybrid Becke, 3-parameter [51] Lee–Yang–Parr functionals [52], and treating 2nd-order scalar relativistic effects within the Douglas–Kroll–Hess (DKH2) [53,54,55] formalism. The calculations were performed with different basis sets: 3-21G or LANL2DZ (Los Alamos National Laboratory 2 double-zeta) for the Ru atom and 6-311++G** or LANL2DZ for all other atoms, or Jorge-TZP (triple-zeta plus polarization)-DKH [56] for all atoms of the molecule, as depicted in Table 2. While all the selected methods yielded symmetrical Ru-N bonds of the same length, the B3LYP-DKH2 formalism with the Jorge-TZP-DKH basis set gave the Ru-N distance (2.079 Å), which is the closest to the value obtained for the RBY(PF_6_)_2_ crystal (2.056 Å) by X-ray diffraction experiments [57]. Since this optimization of the RBY(PF_6_)_2_ system kept the Ru-N bonds and bite angles characteristic of D_3_ symmetry of the RBY ion in the crystal, with the phosphorus anions arranged symmetrically on the C_3_ axis, the same method was consequently applied to optimize the geometry of the other two complexes, RuLp(PF_6_)_2_ and B1(PF_6_)_4_.

For structures of RBY(PF_6_)_2_, RuLp(PF_6_)_2_, and B1(PF_6_)_4_ optimized in their singlet ground states, the vertical transition energies and oscillator strengths for the first 100 singlet excited states were calculated by the TDDFT method at the B3LYP/DKH2 level using the Jorge-TZP-DKH basis set. The energy positions and oscillator strengths were calculated using the iterative Davidson method [58] with an accuracy of 10^−12^ hartree (1 hartree = 4.36·10^−18^ J). Due to the dominant role of singlet excited states in absorption and electroabsorption, only singlet–singlet transitions were in this paper considered, and the SOC effects were neglected. This approximation should be acceptable except for the low-energy tail of the MLCT absorption spectrum where there is a high degree of spin–orbit interaction, and thus, direct excitation of triplet states should be taken into account.

**Figure 2:**
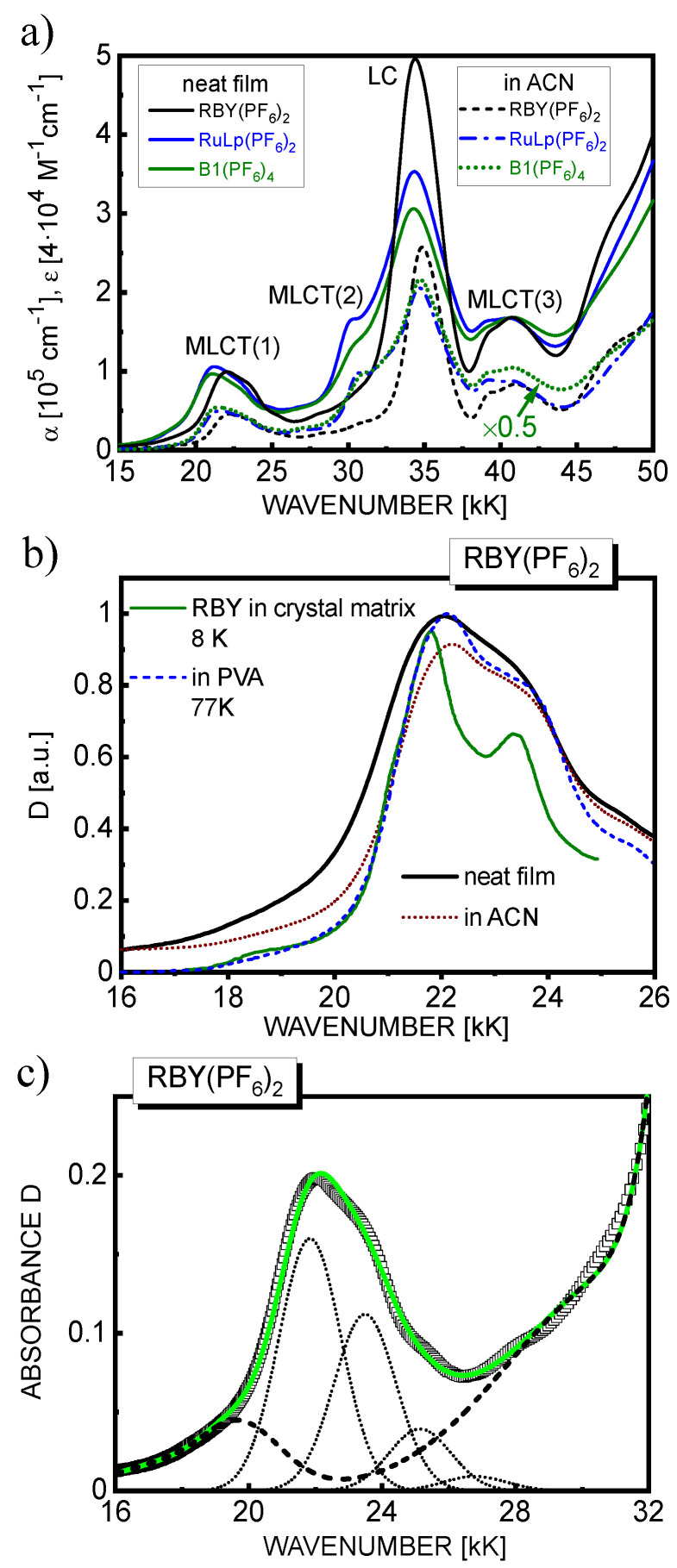
(**a**) Absorption spectra of RBY(PF_6_)_2_, RuLp(PF_6_)_2_, and B1(PF_6_)_4_ in neat films (solid lines) and in ACN solution (broken lines)—for colors, see the website. The molar extinction coefficient (ε) of B1(PF_6_)_4_ was multiplied by 0.5. The spectral ranges of the MLCT and LC excited states are also indicated; (**b**) Comparison of absorption spectra in the MLCT(1) range for RBY(PF_6_)_2_: in a neat film (thick solid line), in an ACN solution (dotted line), in a PVA matrix at 77 K (dashed line, data taken from [30]), and for RBY in the crystal matrix Zn(bpy)_3_(BF_4_)_2_ at 8 K (thin solid line, data from [12]); (**c**) Absorption spectrum of a 46 nm−thick RBY(PF_6_)_2_ film (squares) with the total fit (solid line) and its Franck–Condon components (dotted lines) in the MLCT(1) range (*ν*_0_ = 21.84 kK, Δν = 1.65 kK, *w* = 2.2 kK and *S* = 0.7). The background from the lower energy and higher energy states is represented by a dashed line.

The EA spectra based on TDDFT were calculated by subtracting the absorption spectra without an external electric field from these spectra in the presence of an electric field. Absorption spectra with electric fields oriented along the principal directions (−x, x, −y, y, −z, z) of the molecule were averaged for the isotropic orientation distribution of molecules. In order to compare the theoretical results with the experimental data for solid layers, the Gaussian bands according to Formula (7) were determined, assuming the validity of the Lambert–Beer law. Thus, for the *n*th electronic transition with the TDDFT oscillator strength *f*_n_, the maximal absorbance of the Gaussian band was calculated according to the following formula [7]:

(11) $$D_{0n}=c\;l\;\frac{f_{n}}{4.6\cdot 10^{-9}\;w_{n}}\;,$$

with *c* denoting the molar concentration (in mol/dm^3^), *l* denoting the layer thickness (in cm), and *w*_n_ denoting the bandwidth (in cm^−1^). Taking from the experiment the molar extinction coefficient (ε) of 1.8·10^4^ M^−1^·cm^−1^ for the liquid (ACN) solution and the linear absorption coefficient (α) of 1.0·10^5^ cm^−1^ for the solid film at the main absorption peak of RBY(PF_6_)_2_, we obtained *c* = 2.4 M in good agreement with the RBY(PF_6_)_2_ crystal structure value 2.0 M [49]. Due to similar absorption spectra (Figure 2a), the similar values of *c* were estimated for the other two materials, RuLp(PF_6_)_2_ and B1(PF_6_)_4_.

All of the quantum chemical calculations were performed using the Gaussian 16 package program.

## 4. Results and Discussion

### 4.1. Absorption Spectra

Typical examples of absorption spectra of RBY(PF_6_)_2_, RuLp(PF_6_)_2_, and B1(PF_6_)_4_ in the form of solid neat films with a thickness of 40–50 nm (solid lines) are displayed in Figure 2a in comparison with the absorption spectra of these materials in diluted (10^−5^ M) ACN solutions (broken lines). It can be clearly seen that the absorption spectra of solid layers show almost quasi-identity with the spectra measured in solution. This indicates the absence of excitonic (solid-state) effects and that the solvent shift resulting from the non-resonance interaction between the excited molecule and its solid environment is not significant. Such absorption behavior is very similar to that in solid *fac*-Ir(ppy)_3_ where the electronic excitations delocalized over three ppy ligands are well spatially located within the molecular cage of the complex [28] and therefore rather insensitive for the interaction with the condensed phase environment. However, this is in real contrast to solid films of *mer*-Alq_3_, where in the low-energy part, the ligand-centered (LC) electron excitations localized on one of the quinolate ligands were observed, showing a relatively large (0.4 eV) bathochromic absorption shift from the solution to the solid state in addition to the intermolecular ligand–ligand CT states observed in the high-energy part of the absorption spectrum [27]. 

Based on the well-known classification of RBY excited states [1,7], absorption in the UV range with bands centered at 35 kK (285 nm) and above 45 kK is dominated by the LC π–π* transitions. The two remaining intense bands at 22 kK (450 nm), 42 kK (240 nm), and a shoulder at about 30 kK (330 nm) can be assigned to the MLCT d–π* transitions. In the longwave tail of the first lowest-energy MLCT(1) absorption band, the difficult-to-discern shoulder appears at around 18 kK (550 nm), which is attributed to direct singlet-triplet transitions from ground state to MLCT triplet states induced by strong SOC. 

The absorption properties of the three materials, RBY(PF_6_)_2_, RuLp(PF_6_)_2_, and B1(PF_6_)_4_, are essentially very similar (Figure 2a). The molar extinction coefficient (ε) of the B1(PF_6_)_4_ complex, consisting of two RuLp units, is almost twice as high as that of RBY(PF_6_)_2_ and RuLp(PF_6_)_2_. In the spectral range of MLCT(1) absorption, the maximum value of the linear absorption coefficient (α) is nearly the same and amounts to circa 1.0 × 10^5^ cm^−1^ for solid films of all investigated materials, while a slightly stronger LC absorption (at 35 kK) was shown by solid layers of the RBY(PF_6_)_2_ complex. In addition, some differences in absorption between the functionalized complexes, RuLp(PF_6_)_2_ and B1(PF_6_)_4_, and the parent RBY(PF_6_)_2_ complex can be noticed in the regions of MLCT(1) and MLCT(2) excited states. In comparison to RBY(PF_6_)_2_, the MLCT(1) absorption band is shifted by about 1500 cm^−1^ toward lower photon energies, and the MLCT(2) absorption shoulder at approximately 30 kK is clearly increased in RuLp(PF_6_)_2_ and B1(PF_6_)_4_ complexes. This phenomenon can be ascribed to the contribution of MLCT transitions with an electron transferred to functionalized ligand(s), which is confirmed by TDDFT calculations (see below). Moreover, it was observed that the MLCT(2) band of RuLp(PF_6_)_2_ in the solution strongly responded to the pH changes, which also indicates that the carboxylate-endowed ligand is involved in the corresponding transition [42].

As seen in Figure 2b, the major spectral features of the main MLCT(1) absorption band of RBY(PF_6_)_2_, dominated by singlet excited states, are essentially retained in the liquid or glassy solution, solid layer, and highly symmetric crystalline matrix [Zn(bpy)_3_(BF_4_)_2_] except for phonon-induced spectral broadening when measured at room temperature. The assignment of the electronic states involved in absorption has been constantly discussed in the literature since the first reports on optical absorption of RBY appeared. It is worth surveying only some of the most characteristic mechanisms proposed for the interpretation of the absorption spectrum. The observed absorption peaks were for example ascribed to one MLCT electronic transition, d→π*, which is associated with electron jumps to one or two of the bpy ligands, which are split in energy by electronic interaction with an asymmetrical crystal environment [59]. Other authors prefer to attribute the pronounced spectral features of the MLCT(1) absorption at room temperature to vibronic Franck–Condon progression with a single oscillating mode using for ACN [6] and water [8] solution an average frequency of 1610 cm^−1^ and Huang–Rhys factor S = 0.7, which also reproduce reasonably the absorption spectrum of the solid layer (see Figure 2c). However, there is a variety of experimental and theoretical studies that in general agreement point to a number of different charge transfer states contributing to MLCT absorption in the RBY system. Obviously, these and other mechanisms, such as diverse types of vibrational couplings (adiabatic or non-adiabatic), may most likely cooperate and contribute to the absorption processes at varying rates. 

In the simplest, one-electron excitation model [12,44,60], four frontier molecular orbitals (MO) are considered to be relevant with respect to the number and symmetries of the lowest MLCT transitions (see the orbital energy scheme in Figure 8a). In the D_3_ parent group symmetry, three Ru degenerate t_2g_ orbitals (in the O_h_ point group) due to trigonal distortion energetically split into single a_1_ and doubly degenerate e orbitals. These MOs represent the highest occupied MO (HOMO) and the next-to-HOMO with the energy difference between them being denoted by Δ. The energy level of the antibonding empty π* orbitals of three bpy ligands is also split into single a_2_ and degenerate e terms. These MOs represent the lowest unoccupied MO (LUMO) and the second LUMO, respectively, with an energy difference of Γ. The LUMO with a_2_ symmetry is purely a ligand type, whereas the second LUMO with e symmetry mixes significantly with the next-to-HOMO with the same symmetry. This type of interaction, quantified with the appropriate values of Δ and Γ, results in an additional binding (so-called back-bonding) with extensive delocalization of metal electrons to ligands in the ground state of the complex, which is an important source of ligand–ligand coupling induced by the metal. Accordingly, the greater this orbital mixing, the less net charge transfer occurs upon excitation. Moreover, the LUMO splitting parameter Γ is determined by the electron–electron interaction between the ligands, which can be rationalized as an exciton (resonance) interaction, similar to dendrimer systems (see e.g., [36] and references therein), by analogy with intermolecular excitons (Frenkel type) in molecular crystals [61].

Based on single electron orbital energy scheme (Figure 8a), there are as many as 36 components: 6A_1_, 6A_2_, and 12E can be derived within the MLCT(1) manifold, spanning a range of about 6000 cm^−1^, of RBY electronic system [4]. Although the energetic ordering of the MLCT excited states has not been definitively established and is still discussed in the literature, it has rather been agreed that the highest oscillator strength in absorption is carried by three orbitally degenerate singlet states of total symmetry E, which were polarized perpendicular to the C_3_ axis. Two of them, dominated by $a_{2}\to e$ and e→e electron jumps, deriving their oscillator strength from the “transfer terms”, should be the most intense in absorption [44]. The third one related to the $a_{1}\to e$ transition, nominally of low oscillator strength, may at least in principle derive its intensity from strongly allowed, LC π–π* transitions [60]; however, this mechanism has not yet been well quantified. 

Leaving the one-electron model, the more advanced methods of quantum chemistry such as TDDFT can be used instead. TDDFT gives the energies of the multi-electron excited states, the nature of the filled and virtual orbitals involved in the transitions, and the oscillator strengths associated with these transitions. The TDDFT energy positions of the nine lowest-energy singlet excited states involved in the MLCT(1) absorption of the RBY(PF_6_)_2_ system are comparable with the corresponding results for the RBY ion available in the literature [11,15,62,63]. For comparison, see Table 3, where, next to symmetry symbols for multi-electron transitions, leading single-electron transitions are given in parentheses. Table 3 also shows the TDDFT results, which include a posteriori SOC effects [63]. The splitting parameters Δ and Γ do depend on the degree of electron–electron correlation and exchange interaction, which is one of the reasons for the dependence of the exact positions of the excited state energy levels on the computational levels, such as the forms of electron density functionals. Nevertheless, the energy level scheme for MLCT(1) absorption roughly shares common features. The MLCT(1) absorption area (Figure 8b) includes three E symmetry (in RBY ion) excited states, 1E, 2E, and 3E, prevailingly (*x*,*y*)-polarized, separated by 1000–1700 cm^−1^ with quasi-identical energy positions of their two components in RBY(PF_6_)_2_. The first two lower energy states, 1E and 2E, have close neighbors with A_2_ symmetry, which were polarized along the *z*-direction of the C_3_ axis. The third, highest energy 3E excited state, predominantly e→ e in character, located at about 22.75 kK, is well isolated from other states including also the nominally dipole forbidden state with A_1_ symmetry at 24.93 kK. A characteristic feature of calculations using the TDDFT method is that the most of the MLCT(1) absorption comes from the oscillator strength of the excited state 3E, which is estimated at 0.13. The oscillator strength of the 2E state at 21.09 kK is approximately one order of magnitude lower, and for other states, it is even weaker. The first lowest-energy 1E state at 20.04 kK, predominantly associated with an $a_{1}\to e$ transition, carrying extremely small oscillator strength, does not show any contribution from LC, π→π* transitions; thus, its effect on absorption is rather small according to the TDDFT approach. In addition, the lower-energy tail of the MLCT(1) absorption spectrum is likely to be dominated by directly generated triplet MLCT excited states due to the strong SOC effect, which is currently not included in the common versions of TDDFT calculations. 

The addition of a functional group in RuLp(PF_6_)_2_ distorts the symmetrical properties of the electronic RBY system and thus significantly changes the energy scheme of the excited states and the distribution of their oscillator strengths. While the interaction pattern of the excited state configurations is quite complex, there are some regularities worth noting. The number of excited states that carry noticeable intensity in absorption increases in RuLp(PF_6_)_2_ because the degeneracy of E-type excited states is lifted and some low-intensity A-type states (A_2_ and A_1_ symmetry in RBY ion) gain (or “borrow”) oscillator strengths by interacting with other states. According to the stick-bar chart of the respective oscillator strengths (see Figure 10a), the TDDFT-calculated electronic transitions in RuLp(PF_6_)_2_ can be grouped fairly well and assigned to the MLCT(1), MLCT(2), and LC absorption bands with energy positions and intensities roughly consistent with an experiment. The HOMO–LUMO transition at about 18.9 kK contains Ru-centered HOMO, and LUMO is almost completely localized at a single bpy ligand and its attached carboxyphenyl functional group. The relatively high proportion of unoccupied MOs localized on the part of the molecule containing the functionalized ligand can also be recognized in the first intense low-energy transition of the MLCT(1) absorption band at 20.8 kK and in some transitions belonging to the MLCT(2) band and in the intermediate spectral region of 25–28 kK. It is worth noting here that the energy positions of the most intense LC-type transitions calculated by the TDDFT method are in good agreement with the maximum of the experimental LC absorption band at around 35 kK.

The TDDFT excited states’ interaction pattern is even more complex in the biruthenium B1(PF_6_)_4_ electronic system; however, the main spectral features observed in RuLp(PF_6_)_2_ are still preserved in B1(PF_6_)_4_ (not shown in the figure). This is due to the rather weak electronic coupling between the two RBY units that make up the B1 molecule, which was introduced by the ethynyl-carboxyphenyl linker. Consequently, the MLCT electronic transitions are dominated by the excited state configurations assigned to each RBY unit except that some unoccupied MOs located on the functionalized bpy ligand in RuLp are now located on both bpy ligands linked to the carboxyphenyl functional group. It was also found that the contribution of heterogeneous MLCT transitions related to electron transfer from the Ru centered MO of one RBY unit to the bpy ligands of the other RBY unit is small. The simple energy level scheme can be noticed for the 3E symmetry state at 22.8 kK, which is well distant from others in RBY; this state is degenerate in RBY(PF_6_)_2_, and it is split to two and four components in RuLp(PF_6_)_2_ and B1(PF_6_)_4_, respectively. However, this analysis is more difficult to perform for other states lying close to each other on the energy scale, which may interact and exchange their intensities to varying degrees. Some additional information can be deduced from the study of electroabsorption spectra in the next section.

### 4.2. Electroabsorption Spectra

**Figure 3:**
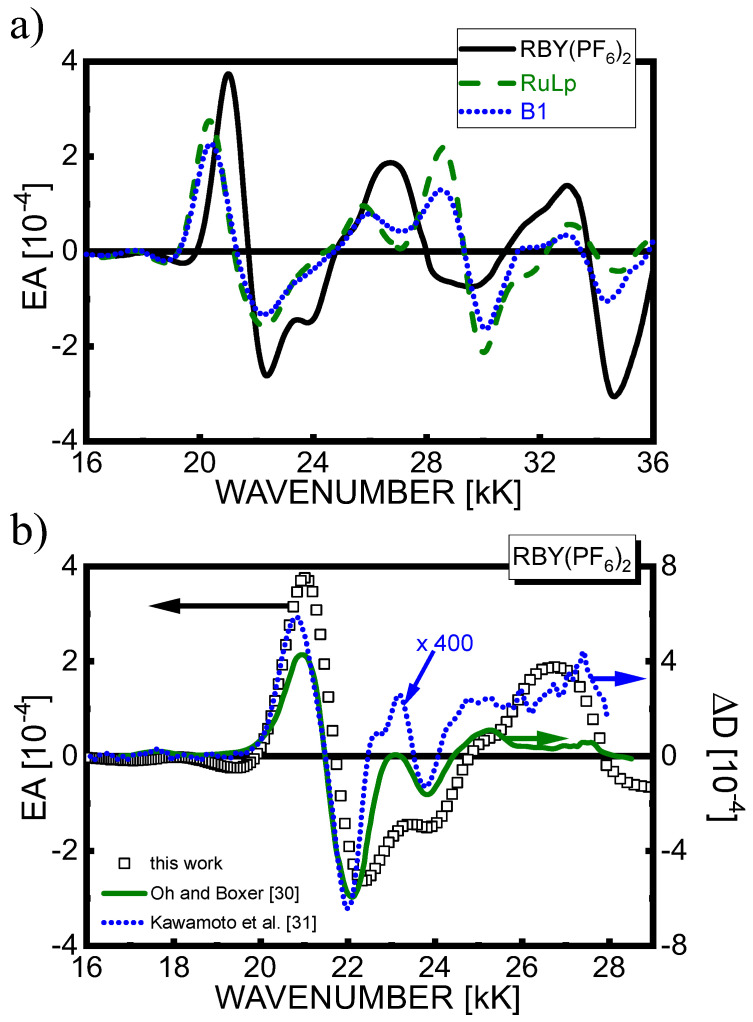
(**a**) Comparison of EA spectra for neat films of Ru(II) complexes: RBY(PF_6_)_2_ (solid line), RuLp(PF_6_)_2_ (dashed line), and B1(PF_6_)_4_ (dotted line); (**b**) Comparison of EA spectrum of a neat film of RBY(PF_6_)_2_ (squares) with those obtained for RBY(PF_6_)_2_ in a PVA matrix at 77 K (solid line, data taken from [30]) and RBY(Cl)_2_⋅6H_2_O in PVA at room temperature (dotted line, data from [31]).

The EA spectra for Al/Ru complex film/Al samples in an external electric field *F*_rms_ = 6–7·10^5^ V/cm, displayed in Figure 3a in the spectral range 16–36 kK including MLCT(1), MLCT(2), and LC excited states, exhibit the behavior consistent with their absorption spectra in Figure 2a. The EA spectra of RuLp(PF_6_)_2_ and B1(PF_6_)_4_ are essentially very similar in terms of signal shape and magnitude. In relation to the corresponding EA spectrum of RBY(PF_6_)_2_, they show some differences in the ranges: 20–22 kK (with the redshift of the positive low-energy EA lobe) and 25–30 kK (with rather dissimilar lineshapes), i.e., in spectral regions where excited states associated with functionalized bpy ligands were indicated (see Section 4.1). As shown in Figure 3b, our EA spectra in the MLCT(1) absorption range recorded for neat RBY(PF_6_)_2_ films using non-polarized light agree very well with the previous ones obtained using a magic angle (χ = 54.7°) polarized light beam for RBY(PF_6_)_2_ and RBY(Cl)_2_⋅6H_2_O dispersed in a polymer PVA matrix, by Oh and Boxer at 77 K [30], and by Kawamoto et al. at room temperature [31]. The more than two orders of magnitude lower Δ*D* signals reported by Kawamoto et al. are due to at least one order less electric field applied to their samples with an interdigitated arrangement of surface electrodes.

**Figure 4:**
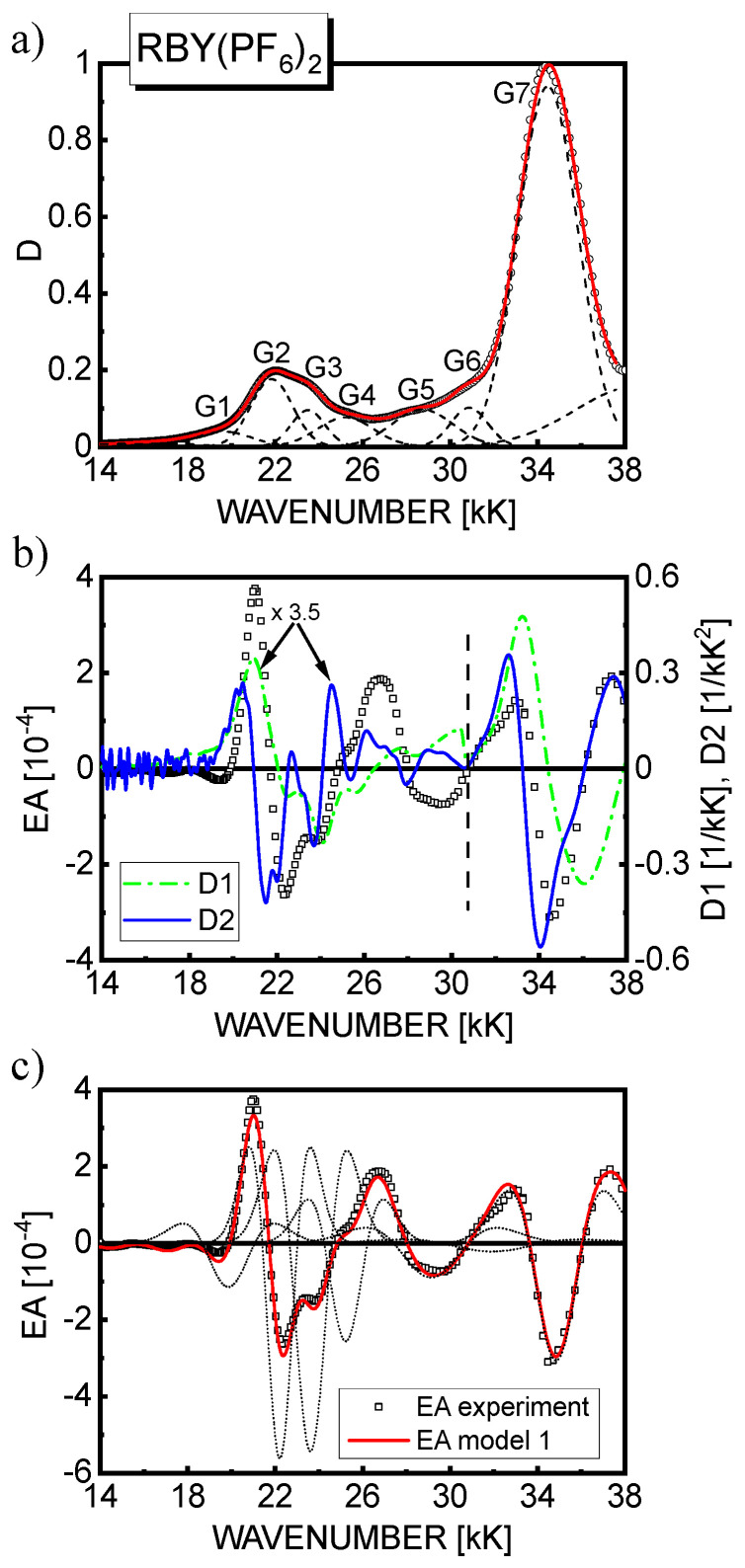
(**a**) The absorption spectrum of a 46 nm−thick RBY(PF_6_)_2_ (squares) and its decomposition into Gaussian bands. The solid line obtained by the superposition of the Gaussian profiles (dashed lines); (**b**) The EA spectrum of the RBY(PF_6_)_2_ film (squares) measured at an electric field strength with the rms value, *F*_rms_ = 7.3·10^5^ V/cm, compared with the calculated first (D1, dash−dotted line) and second (D2, solid line) derivatives of the absorption spectrum displayed in part (a) of the figure. The signal values of all spectra were multiplied by a factor of 3.5 in the lower−energy range indicated by the dashed vertical line; (**c**) The best fit of experimental EA spectrum of RBY(PF_6_)_2_ film (squares) with the theoretical curve calculated according to model 1 (solid line). The dotted lines represent the second derivative contributions to the EA signal from the relevant Gaussian absorption bands.

**Figure 5:**
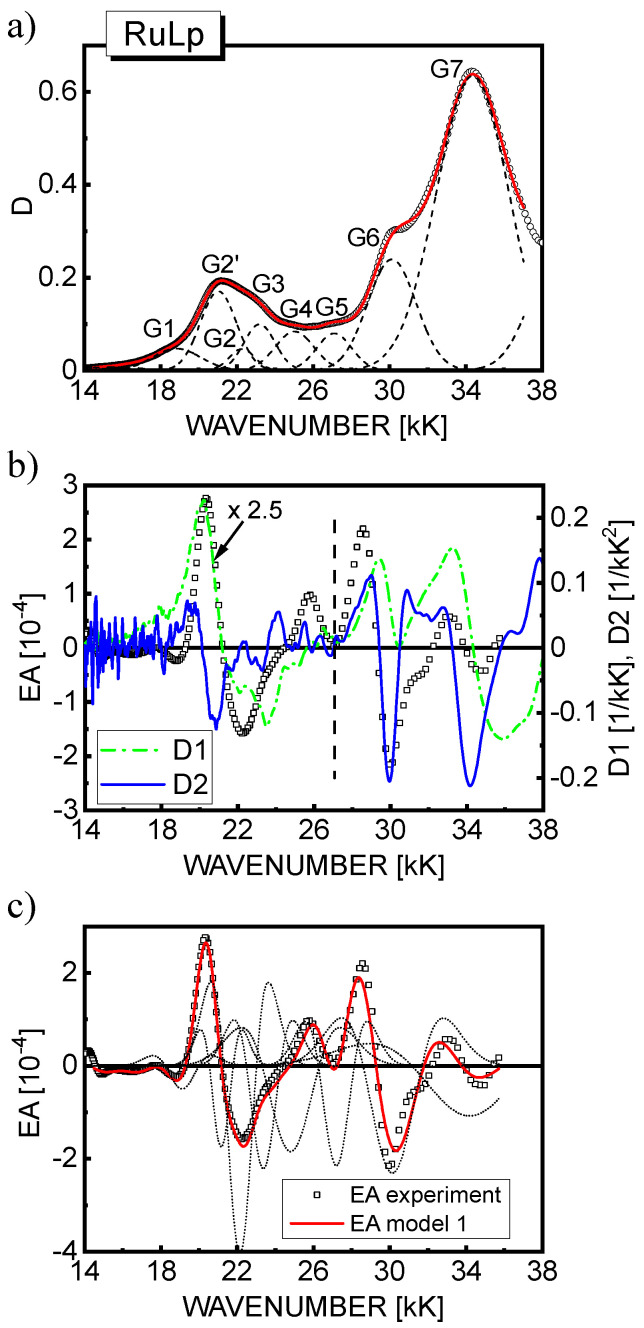
(**a**) The absorption spectrum of a 42 nm−thick RuLp(PF_6_)_2_ (squares) and its Gaussian profile analysis. The solid line obtained by the superposition of the Gaussian profiles (dashed lines); (**b**) The EA spectrum of RuLp(PF_6_)_2_ film (squares) measured at the electric field strength, *F*_rms_ = 6·10^5^ V/cm, compared with the calculated first (D1, dash−dotted line) and second (D2, solid line) derivatives of the absorption spectrum shown in part (a) of the figure. The values of all signals were multiplied by a factor of 2.5 in the lower−energy range indicated by the dashed vertical line; (**c**) The best fit of experimental EA data for RuLp(PF_6_)_2_ (squares) with the theoretical curve calculated according to model 1 (solid line). The dotted lines represent the second derivative contributions from the corresponding Gaussian profiles.

**Figure 6:**
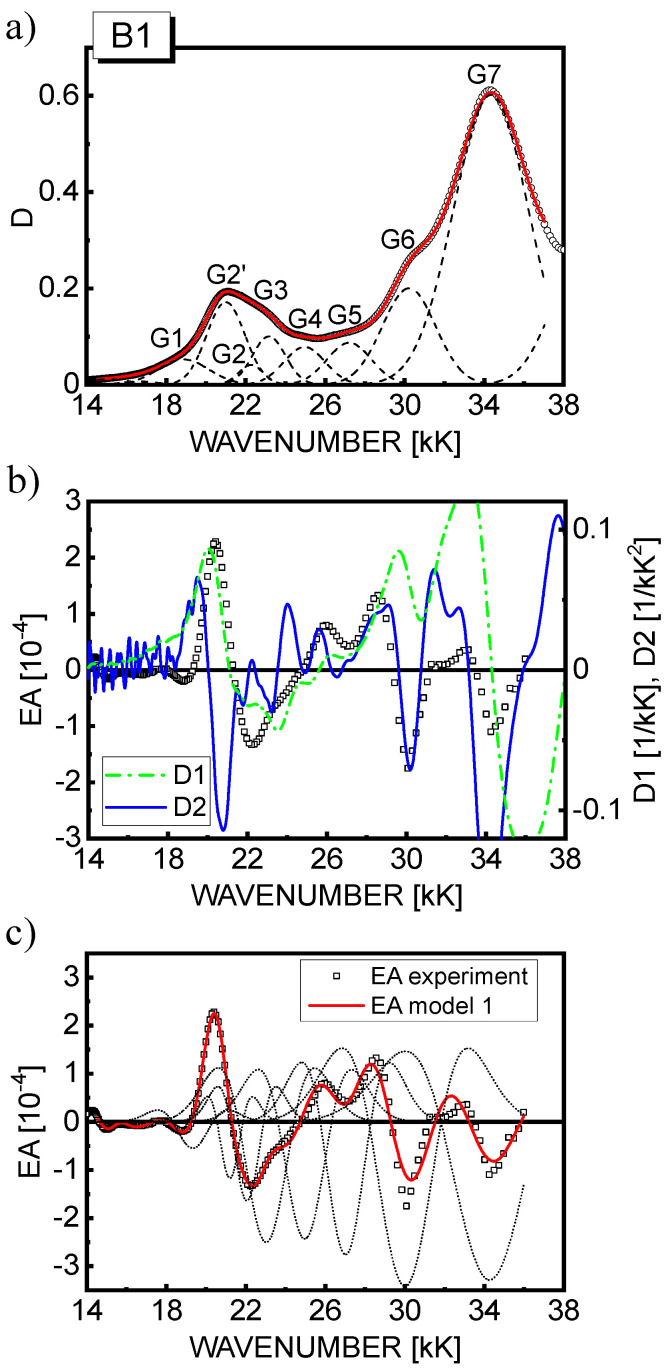
(**a**) The absorption spectrum of a 46 nm−thick B1(PF_6_)_4_ (squares) and its Gaussian profile analysis. The solid line was created by the superposition of the Gaussian profiles (dashed lines); (**b**) The EA spectrum of B1(PF_6_)_4_ film (squares) recorded at the electric field, *F*_rms_ = 5.7·10^5^ V/cm, compared with the calculated first (D1, dash−dotted line) and second (D2, solid line) derivatives of the absorption spectrum shown in part (a) of the figure; (**c**) The best fit of experimental EA spectrum of B1(PF_6_)_4_ film (squares) with the theoretical curve calculated according to model 1 (solid line). The dotted lines represent the second−derivative contributions from the corresponding Gaussian profiles.

**Figure 7:**
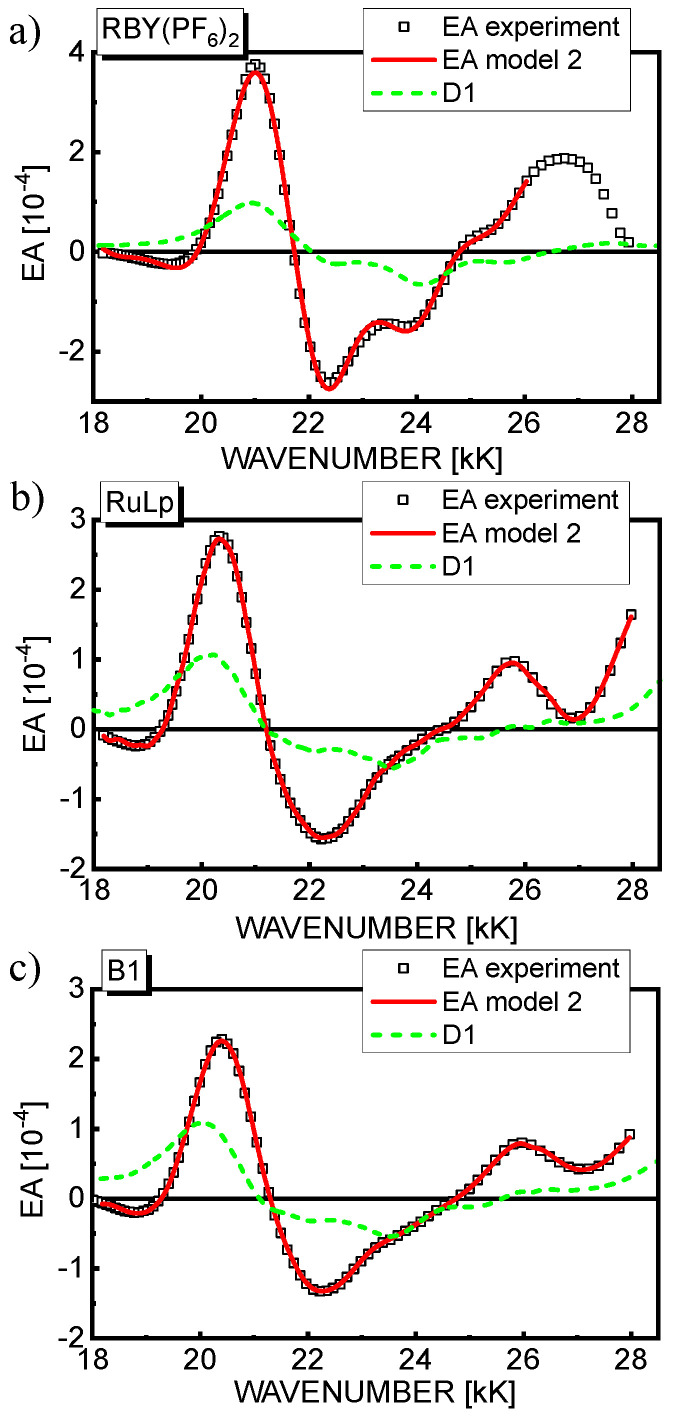
The best fits of EA spectra, taken from Figure 4, Figure 5 and Figure 6, obtained on the basis of model 2 for the films of Ru(II) complexes: RBY(PF_6_)_2_ (**a**), RuLp(PF_6_)_2_ (**b**), and B1(PF_6_)_4_ (**c**). The contribution of the first derivative (D1) of the respective global absorption spectrum to the EA signal (see Equation (9)) is shown by the dashed line.

A judicious discrete parametrization and fitting of the EA spectra using the models described in Section 2 was carried out self-consistently with the decomposition of the absorption spectra into a sum of several Gaussian profiles, as displayed in Figure 4, Figure 5, Figure 6 and Figure 7. Accordingly, the Gaussian components can be ascribed to the relevant excited state spectral regions. The MLCT(1) absorption range includes components G2, G2′ (in RuLp and B1 complexes), G3, and G4; MLCT(2) absorption is formed by component G6; and the most intense high-energy LC absorption belongs to component G7. Moreover, the G5 and partially G4 components are related to the intermediate MLCT region, and the broad G1 band in the lower energy tail of the absorption spectra can be ascribed to the direct triplet MLCT(1) absorption. As shown in part (b) of Figure 4, Figure 5 and Figure 6, the shape of EA spectra only roughly resembles the second derivative of global absorption spectra (D2, solid line), but a certain contribution of the first derivative spectrum (D1, broken line) especially in the MLCT(1) excited states range can also be noticed. Based on the global D2 derivative spectrum, for RBY(PF_6_)_2_ in the PVA matrix, Oh and Boxer [30] estimated the dipole moment change Δμ of 8.8 D/*f* and 5.3 D/*f* in the lower and higher energy parts of the MLCT(1) region, respectively. In turn, using Equation (8), Kawamoto et al. [31] obtained an Δμ of 3.3 D/*f*, 6.7 D*/f*, and 5.8 D/*f* for the three Gaussian components extracted from the MLCT(1) absorption.

Based on their RBY(Cl)_2_:PVA samples, the value of the field correction factor *f* is close to 1.0, which is estimated at 1.1–1.3 for polymer PVA or glassy samples [22,64]. However, for the solid organic neat films of Ru complexes considered here, this factor should be rather close to 2.0, as estimated in the Lorentz field approximation for Alq_3_ vacuum evaporated layers [27]. 

Based on model 1, a reasonably good fit of the theoretical EA curves calculated according to Equation (8) (solid line) with the experimental EA data (squares) is achieved as displayed in part (c) of Figure 4, Figure 5 and Figure 6 for the respective materials. For completeness, the contributions to the EA signal of the second derivatives of the individual *n*th Gauss components are also shown (dotted lines). In the spectral region 18–28 kK, where the first-derivative D1 contribution is predicted, the fitting procedure was repeated applying model 2, and as seen in Figure 7, the fit quality of the theoretical EA spectra (solid lines) calculated according to Equation (9) is even better than in model 1. Although the EA signal is really dominated by the terms with the second derivative in Equation (9), the contribution of D1 to the EA spectra (dashed lines in Figure 7) in the range of MLCT(1) absorption (18–25 kK) appears not to be negligible, as previously assumed for RBY systems [30,31]. From the values of fitting parameters, *b_n_* and *a*, assuming the averaging factor κ = 1/3 (confer Table 1) and taking the *D*_0*n*_ amplitudes of the relevant Gaussian bands, the changes of dipole moments Δμ*_n_* were calculated, and in the case of model 2, the average change of electron polarizability Δ*p* was calculated as well, which is summarized in Table 4 for all three complexes. It is worth noting that it is difficult to extract the absolute magnitudes of *D*_0*n*_ from the absorption spectrum in a unique way, especially in the MLCT(1) range, where four or five Gaussian components are hidden under the total absorption envelope. Therefore, the Δμ*_n_* parameters were calculated in two ways: firstly, on the basis of the amplitudes *D*_0*n*_ taken from the Gaussian bands displayed in part (a) of Figure 4, Figure 5 and Figure 6, then assuming that the amplitudes are equal to the total absorbance *D* in the global absorption spectrum. The values of Δμ*_n_* obtained by the latter method are presented in Table 4 in parentheses for both models. 

In the case of RBY(PF_6_)_2_ layers, the average value of Δμ*_n_* in the singlet MLCT(1) absorption range is Δμ_av_ = 7–9 D/*f* according to both models with Δ*p* = 40 Å^3^/*f*^2^ (1 Å^3^ = 1.11·10^−40^ F·m^2^), as estimated in model 2. Using the typical value of the local field correction factor *f* = 1.7 for organic molecular films, the corrected value of Δμ_av_’ = 4–5 D correlates well with the average values of Δμ obtained earlier for RBY:PVA systems in the same spectral range [30,31]. In the case of RuLp and B1 complexes, according to model 1, Δμ_av_ = 8–11 D/*f* is similar for both types of complexes and slightly larger than in RBY(PF_6_)_2_, as expected for unsymmetrical molecules with polar functional groups. However, this relationship is inverted on the basis of model 2, where Δμ_av_ = 5–7 D/*f* is slightly lower than in RBY(PF_6_)_2_; hence, no unambiguous conclusion can be drawn from it. The contribution of the G1 band to the EA fitting curve in the low-energy tail of the absorption spectrum is rather relatively small in all three complexes. It is well known that this low absorbance region can be modified by light scattering; therefore, due to the high uncertainty in determining the absolute absorption intensity of triplet MLCT(1) states buried under the G1 profile, reliable values of Δμ were difficult to determine, and this spectral region will not be analyzed further. The polarizability change upon excitation Δ*p*, according to model 2, for the RuLp(PF_6_)_2_ complex containing a functionalized RBY unit is Δ*p* = 70 Å^3^/*f*^2^, and for the B1(PF_6_)_4_ complex containing two RBY units, it is Δ*p* = 90 Å^3^/*f* ^2^, the value of which is about twice as large as in the RBY system. The Δ*p* results seem highly realistic when compared with the average polarizability Δ*p* = 20 Å^3^/*f*^2^ for the first five MLCT transitions in Ir(ppy)_3_ films [28] and Δ*p* = 60 Å^3^/*f*^2^ for the first five low-energy LC transitions in Alq_3_ films [27]. Therefore, it can be conceived that the electron polarizability in such complexes is mainly determined by electron-rich π-conjugated ligands.

Further regularities can also be noted for the higher-energy excited states of MLCT(2) and LC origin, based on the electric field effect on G6 and G7 Gaussian bands, respectively. In RBY(PF_6_)_2_, the change in dipole moment for LC transitions Δμ_7_ = 3.3 (3.5) D/*f* is slightly lower than Δμ_6_ = 5.3 (4.5) D/*f* for MLCT(2) and two to three times lower than Δμ_av_ for the MLCT(1) spectral range. This trend in the differences between Δμ_7_, Δμ_6_, and Δμ_av_ fades coming from RBY to RuLp and B1, with Δμ_7_ = 7.5 D/*f* in the RuLp complex and Δμ_7_ = 12 (11) D/*f* in the B1 complex. As confirmed by TDDFT calculations, the greater values of Δμ_7_ and Δμ_6_ in RuLp and B1 than in RBY can be attributed to longer-range electron jumps to more delocalized π* ligand orbitals that extend into regions conjugated to the ethynyl-carboxyphenyl functional group. As this feature also belongs to the unoccupied π* orbitals involved in the spectral range of the G4 and G5 bands, the higher values of Δμ_4_ and Δμ_5_ in RuLp and B1 than in RBY can be similarly explained.

**Figure 8:**
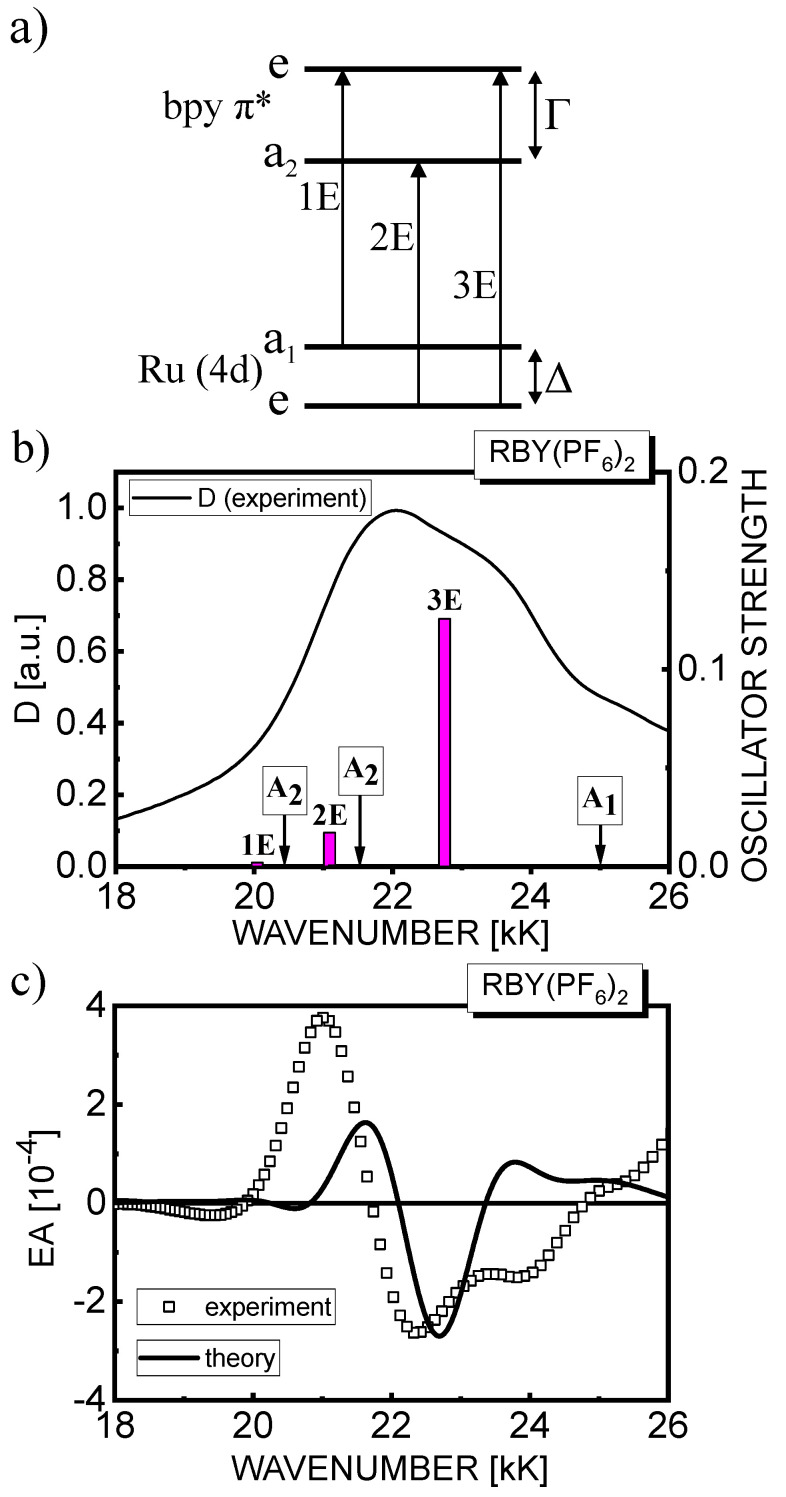
(**a**) Simplified diagram of single−electron energy levels for RBY complex in D_3_ symmetry (based on [44,60]). The splitting between the levels of the filled 4d Ru orbitals is denoted by Δ, and that between the levels of the empty ligand π* orbitals is denoted by Γ. The three doubly−degenerate MLCT transitions (1E, 2E, and 3E) are also indicated; (**b**) Stick−bar spectrum of the calculated oscillator strengths using the TDDFT method for RBY(PF_6_)_2_ in a vacuum superimposed on the experimental absorption spectrum of the RBY(PF_6_)_2_ film; (**c**) The EA spectrum calculated using the TDDFT method (solid line) for all excited states presented in part (**b**) of the figure compared with the experimental EA spectrum of the film (squares).

**Figure 9:**
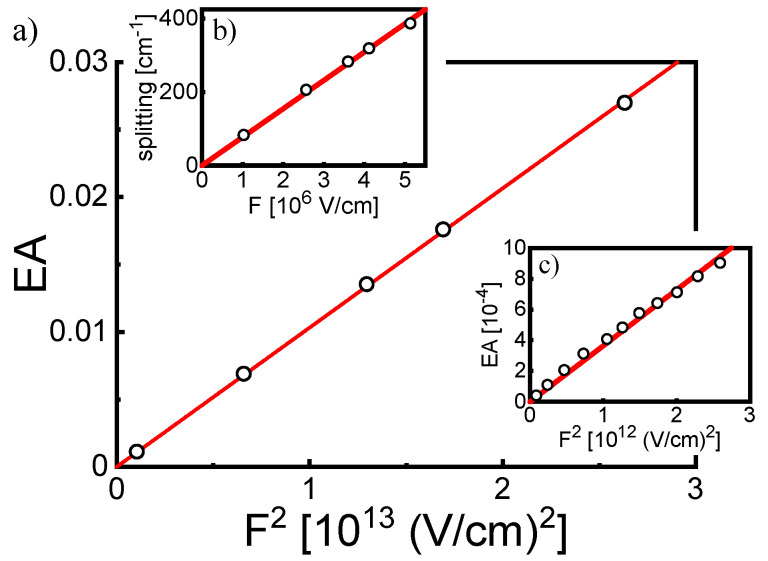
Behavior of the 3E state of the RBY(PF_6_)_2_ system in the electric field. The electric field dependence of the EA signal calculated by the TDDFT method is shown in part (**a**) on the 10^6^ V/cm field scale, and for the experimental EA signal, it is shown in part (**c**) on the 10^5^ V/cm field scale. The graphs are linear with the second power of the applied electric field strength. Part (**b**) of the figure shows the linear dependence on the electric field of splitting the two components of the 3E state, as calculated by the TDDFT method.

As discussed in Section 3, the local symmetry of the RBY ion in the RBY(PF_6_)_2_ solid films is most likely close to the D_3_ symmetry of this ion in an ideal crystal environment [49]. In addition, the DFT optimized molecular structure of the RBY(PF_6_)_2_ system (in a free space) retains the basic geometric properties of the D_3_ point group. An inherent feature of D_3_ symmetry is the degenerate E-type electronic states and in fact, according to our TDDFT results, the MLCT(1) area in both absorption (Figure 8b) and electroabsorption (Figure 8c) is dominated by the excited state 3E at position 22.75 kK. The TDDFT values of the EA signals were calculated as described in Section 3.3 assuming the bandwidth *w*_n_ = 1.35 kK for all excited states, which is by a factor of 1.5 smaller than the widths obtained in the fitting procedure using Equation (8) or (9). The EA theoretical curve (solid line) only roughly resembles the shape of the EA experimental spectrum in Figure 8c, but the TDDFT calculations probably underestimate the oscillator strengths of other E-states (1E and 2E) predicted in the MLCT(1) range, which can be seen in the zero-field absorption spectrum in Figure 8b. In order to attain reliable EA signals that are definitely greater than the numerical noise, TDDFT calculations were performed at electric field strength of several 10^6^ V/cm, i.e., one order of magnitude higher than the applied experimental electric field. Therefore, the values of experimental EA signals in Figure 8c are about two orders lower than the theoretical values, which is consistent with the electroabsorption mechanism following the Stark effect (see Formula (4)). The validity of this behavior of EA is illustrated by the linear dependence of the maximum EA signals (at 22.69 kK) on the square of the applied electric field for both TDDFT results, as shown on the 10^6^ V/cm scale in Figure 9a, and the experimental results shown on the 10^5^ V/cm scale in Figure 9c. The important message from the TDDFT computation results is that the second derivative-like shape of EA response in Figure 8c is mainly governed by the first-order Stark effect in the doubly degenerate state 3E. The energy splitting (Δ*E*) of the two sub-components of the 3E state is exactly linear with the electric field (Figure 9b), which allows determining the dipole moment matrix element μ_12_ = Δ*E*/2*F* ≅ 2.5 D. These two sub-components of the 3E state lie in the inhomogeneously broadened absorption spectrum, together giving the EA signal similar to the second absorption derivative. Using the Formulas (10) and (11) for an isolated Gaussian band with a width *w* = 1.35 kK, the dipole moment value of 4.9 D was obtained for the TDDFT curve in Figure 8c, which correlates well with Δμ_av_’ = 4–5 D estimated above for the MLCT(1) spectral range by curve fitting to the Equation (8) in Figure 4c. The value of the dipole moment is about twice as large as that resulting from the analysis of the splitting of the degenerate state, but the approximation ignores the influence of other electronic states, assuming the shape of the EA spectrum as the second derivative of a single absorption band. For comparison, the dipole moments of E-type states (in the C_3_ point group) identified by a similar method in the lowest-energy absorption band in triphenylamine (TPA) are about 4 D, and in the *m*-MTDATA dendrimer consisting of three TPA branches, they are about 8 D [36], which suggests a slightly stronger exciton coupling between TPA units in the *m*-MTDATA molecule than between the bpy ligands in the RBY complex. According to TDDFT results, the splitting pattern of two other degenerate states (1E and 2E in Figure 8b), due to the closely distant A_2_ states, is more complicated; in addition, the symmetry forbidden A_1_ state (at about 25 kK) acquires a certain intensity in the electric field. However, at the current level of TDDFT computation, these states are endowed with relatively low oscillator strengths, and therefore, as can be seen in Figure 8c, they modify the second derivative-like EA signal from the dominant state 3E in a fairly moderate manner.

We emphasize here that in order to interpret the EA behavior of RBY(PF_6_)_2_ solid films, there is no need to invoke the charge localization model. According to this model, previously applied to RBY systems [30], the anticipated change Δμ in the dipole moment was attributed not to the degeneracy of excited states but to the transfer of an electron from the Ru metal center to a single bpy ligand. However, the asymmetric charge redistribution in a molecule having a ground state with D_3_ symmetry requires the crude assumption of breaking the symmetry of the molecule in Franck–Condon states. While environment-induced Franck–Condon state distortion can be envisaged in disordered liquid or glassy systems, the present EA results for the solid neat RBY(PF_6_)_2_ films with possible D_3_ site symmetry for the RBY ions can be reasonably well interpreted as discussed above without assuming such distortion in the excited state.

**Figure 10:**
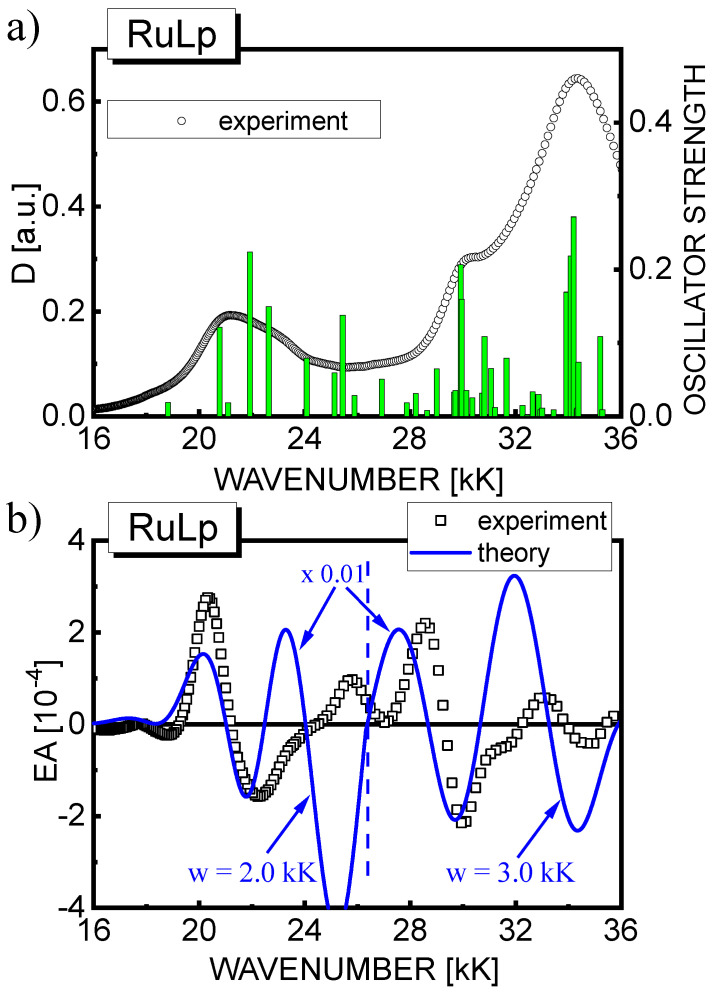
(**a**) Stick−bar spectrum of the calculated oscillator strengths using the TDDFT method for RuLp(PF_6_)_2_ in a free space overlaid on the experimental absorption spectrum of the RuLp(PF_6_)_2_ film (circles); (**b**) The EA spectrum calculated using the TDDFT method for all excited states presented in part (**a**) of the figure (solid line) compared with the experimental EA spectrum of the film (squares). The EA signals were calculated assuming the same Gaussian bandwidths: *w* = 2 kK for all excited states in the lower energy range and *w* = 3 kK in the higher energy range, as indicated by the vertical dashed line separating the two areas.

As discussed in Section 4.1, in the RuLp(PF_6_)_2_ system, which is characterized by an asymmetric molecular structure, there are no strictly degenerate states, but many electronic states closely lying on the energy scale interact with each other (Figure 10a). The cumulative EA response from all of these states determined by the TDDFT method (solid line) is compared with the experimental results (squares) in Figure 10b. The TDDFT curve assumes the average bandwidths taken from Figure 5a, *w* = 2 kK and *w* = 3 kK, for the lower and higher energy ranges, respectively, as indicated in the figure by a dashed vertical line. As in the case of RBY(PF_6_)_2_, the EA spectrum was calculated for an electric field strength (5·10^6^ V/cm) one order of magnitude higher than the applied experimental electric field; therefore, the EA signals are two orders larger than the measured ones. Apart from the intense negative lobe associated with MLCT states at about 25 kK, which is not seen at all in the experiment, the theoretical curve in Figure 10b somehow, while not fully satisfactory, reproduces the main features of the experimental EA spectrum. A structure similar to the second derivative, with a negative minimum at about 22.0 kK, is mainly formed by the 3E state (in RBY) now Stark-split into two components by an intramolecular electric field and slightly modified by other MLCT(1) states endowed with more pronounced oscillator strengths than in the RBY system. The value of the dipole moment parameter Δμ = 5 D estimated by Formula (10) is approximately the same as in the RBY system, which proves the resistance of the electronic MLCT(1) system to environmental disturbances, as previously noted [9,15]. 

A closer look at the TDDFT results in Figure 10b shows that the total EA signal consists of a series of components of individual excited states that add up in some spectral ranges and compensate in others. This collective nature of the EA response, especially in high-energy spectral ranges with densely packed excited states, makes it impossible to practically determine the molecular parameters for the individual excited states, at least on the basis of the analysis of EA spectra measured under broadband spectroscopy conditions. Therefore, in the case of a dense manifold of excited states, a discrete parameterization of EA spectra in terms of Δμ and Δ*p* parameters, such as that carried out in Table 4 for RBY, RuLp, and B1 complexes, except for spectral regions with well-isolated excited states, is not very informative. It can be regarded as a technical method comparing the average EA responses of materials under study rather than as a direct insight into electronic processes just after photoexcitation. We add here that the TDDFT method enables modeling of the EA spectra taking into account the collective response of all excited states involved in the spectrum without restrictions imposed by the lineshape of the EA signal, although as shown in Figure 8c and Figure 10b, the current level of TDDFT calculations does not allow for a satisfactory reproduction and interpretation of EA results for the Ru complexes analyzed in this article. In order to better reproduce EA spectra, the strong SOC effect and the various pathways of vibronic coupling, as mentioned in the Introduction, should certainly also be included in more sophisticated models.

## 5. Conclusions

Electronic states of tris(bipyridine) Ru(II) complexes were investigated by analyzing the electroabsorption spectra of the solid neat films of RBY(PF_6_)_2_, RuLp(PF_6_)_2_, and B1(PF_6_)_4_. Our EA results for neat solid RBY(PF_6_)_2_ films are very similar with the literature data for RBY(PF_6_)_2_ and RBY(Cl)_2_⋅6H_2_O species dissolved in glassy or polymer, naturally more disordered, environments, which indicates at resistance of the electronic MLCT(1) system of RBY species to environmental interactions. Standard analysis of EA spectra based on absorption derivatives does not provide unique sets of dipole moment parameters for the studied complexes, especially in the spectral ranges with densely packed excited states. Therefore, the EA spectra of RBY(PF_6_)_2_ and RuLp(PF_6_)_2_ systems were compared for the first time with the spectra computed by the TDDFT method without making any additional assumptions about the spectral lineshape of the EA signal. We have shown that the main EA feature, in a form close to the absorption second derivative, observed in the spectral range of the first MLCT absorption band in RBY(PF_6_)_2_, can be attributed to a delocalized and orbitally degenerate excited state. This observation, although taken for solid RBY(PF_6_)_2_ layers, may also have key implications for the EA mechanism in other RBY-based systems that exhibit similar EA spectra, due to the robust nature of MLCT(1) electronic states in such systems. Thus, the EA spectrum revealing the shape of the second derivative of absorption band cannot be taken as direct evidence of the permanent dipole moment in the Franck–Condon excited state for a molecule with D_3_ symmetry, unless there is an asymmetric environment-induced distortion of the molecule. Although such distortions can be conceivable in liquid and glassy systems [30,31], they are less likely to occur in the solid RBY(PF_6_)_2_ layers investigated here. Moreover, as discussed in the Introduction, there are many examples provided by ultrafast spectroscopy experiments, which show that the distortion of a molecule in the Franck–Condon state starts after the primary photoexcitation event and evolves over a longer time scale.

As RuLp and B1 complexes have recently been successfully applied as photosensitizers in DSSCs [41,42], we plan to extend our EA spectroscopic studies with molecules of complexes anchored on the TiO_2_ surface, as in real DSSC devices. Comparison of the EA spectra of such systems with and without the TiO_2_ semiconductor will allow to determine the contribution of TiO_2_ ionic states to the primary excited states involved in the charge separation process, which limits the efficiency of photocells and has so far been used for the N719 dye [65], and such studies are underway.

## Figures and Tables

**Table 1:** Averaging factors κ for non-degenerate and degenerate states.

Electronic State	κ	Ref.
Non-degenerate state	$\frac{1}{3}$	[43]
	$\frac{1}{5}$	[24]
Doubly degenerate state	$\frac{3}{10}$	[34]
	$\frac{1}{3}$	[36]

**Table 2:** The distances (in Å) between the Ru ion and neighboring N atoms for a RBY(PF_6_)_2_ molecular system calculated by different DFT methods.

Method		DFT/B3LYP	DFT/B3LYP	DFT/B3LYP	DFT/B3LYP-DKH2
Basis set	Ru	3-21G	LANL2DZ	LANL2DZ	Jorge-TZP-DKH
	Other atoms	6-311++G**	6-311++G**	LANL2DZ	Jorge-TZP-DKH
Ru-N		2.112	2.100	2.096	2.079

**Table 3:** Wavenumbers (in cm^−1^) for vertical MLCT transitions in RBY calculated by the TDDFT method.

Transition	Heully et al. [63]		Stark et al. [15]	This Work
	without SOC	with SOC	without SOC	without SOC
$A_{2}\;\left(a_{1}\to a_{2}\right)$	19,929	19,487	21,786	19,595
$E\;\left(a_{1}\to e\right)$	20,256	21,004	22,119	20,043/44
$A_{2}\;\left(e\to e\right)$	21,729	21,842	23,998	21,155
$E\;\left(e\to a_{2}\right)$	21,817	22,015	23,855	21,088/90
E (e→e)	23,322	23,411	24,722	22,747/50
$A_{1}\;\left(e\to e\right)$	25,120	25,195	26,344	24,929

**Table 4:** Results of fitting model parameters for EA spectra of Ru(II) complexes. The uncertainty of parameters Δμ and Δ*p* is around 10%.

MATERIAL	BAND	ABS	Model 1			Model 2			
		Posit.	Posit.	*f·*Δμ	(*f·*Δμ)_av_	Posit.	*f·*Δμ	(*f·*Δμ)_av_	*f*^2^ Δp
		[kK]	[kK]	[D]	[D]	[kK]	[D]	[D]	[Å^3^]
RBY(PF_6_)_2_	1	19.65	19.87	10 (8.1)		19.97	9.7 (7.5)		40
	2	21.84	22.22	7.1 (6.7)	9.0 (7.9)	22.16	6.5 (6.1)	8.3 (7.0)	
	3	23.50	23.62	11 (8.8)		23.63	11 (8.1)		
	4	25.21	25.23	9.0 (8.2)		25.30	7.4 (6.9)		
	5	28.73	29.14	8.2 (8.3)		28.01	10 (10)		
	6	30.88	31.92	5.3 (4.5)					
	7	34.47	34.85	3.3 (3.5)					
RuLp(PF_6_)_2_	1	18.85	19.12	5.5 (4.7)		19.19	12 (9.8)		70
	2’	21.04	21.17	3.8 (3.6)	10 (7.6)	21.20	3.2 (3.1)	7.0 (5.1)	
	2	22.28	22.14	16 (7.9)		22.12	11.9 (6.1)		
	3	23.19	23.37	8.0 (6.7)		23.14	6.8 (5.5)		
	4	25.03	24.81	13 (12)		24.60	6.1 (5.6)		
	5	27.11	27.20	9.3 (8.2)		27.06	7.4 (6.5)		
	6	30.13	30.14	9.1 (8.1)					
	7	34.36	34.18	7.5 (7.5)					
B1(PF_6_)_4_	1	18.87	19.33	6.6 (5.1)		19.31	12 (10)		90
	2’	21.00	21.20	3.4 (3.2)	11 (8.1)	21.26	2.9 (2.8)	7.6 (5.4)	
	2	22.27	22.06	11 (5.1)		22.12	12 (5.5)		
	3	23.17	23.03	14 (11)		23.16	8.0 (6.6)		
	4	24.96	24.97	15 (13)		24.80	7.5 (6.6)		
	5	27.21	27.02	14 (13)		27.10	7.0 (6.2)		
	6	30.22	30.00	15 (13)					
	7	34.34	34.26	12 (11)					

## Data Availability

Not applicable.
